# Comparative proteomics between natural Microcystis isolates with a focus on microcystin synthesis

**DOI:** 10.1186/1477-5956-10-38

**Published:** 2012-06-07

**Authors:** Ângela Tonietto, Bernardo A Petriz, Wérika C Araújo, Ângela Mehta, Beatriz S Magalhães, Octávio L Franco

**Affiliations:** 1 Centro de Análises Proteômicas e Bioquímicas, Universidade Católica de Brasília, Pós Graduação em Ciências Genômicas e Biotecnologia, SGAN 916 Norte Av. W5, Brasília, DF, Brazil; 2 Embrapa Recursos Genéticos e Biotecnologia, Brasília, DF, Brazil; 3 Departamento de Biologia, Universidade Federal de Juiz de Fora, Juiz de Fora, MG, Brazil

**Keywords:** Cyanobacteria, *Microcystis aeruginosa*, Cyanotoxin, Proteome

## Abstract

**Background:**

*Microcystis aeruginosa* is a species of cyanobacteria commonly found in a number of countries and frequently related to animal poisoning episodes due to its capacity to produce the cyanotoxin known as microcystin. Despite vast literature on microcystin structures and their deleterious effects, little is known about its synthesis by cyanobacteria. Therefore, this study used proteomic tools to compare two *M. aeruginosa* strains, contrasting them for microcystin production.

**Results:**

2-DE gels were performed and 30 differential protein spots were chosen. Among them, 11 protein spots were unique in the toxin producing strain and 8 in the non-toxin producing strain, and 14 protein spots were shown on both 2-DE gels but expressed differently in intensity. Around 57% of the tandem mass spectrometry identified proteins were related to energy metabolism, with these proteins being up-regulated in the toxin producing strain.

**Conclusions:**

These data suggest that the presence of higher quantities of metabolic enzymes could be related to microcystin metabolism in comparison to the non-toxin producing strain. Moreover, it was suggested that the production of microcystin could also be related to other proteins than those directly involved in its production, such as the enzymes involved in the Calvin cycle and glycolysis.

## Background

*Microcystis aeruginosa,*a worldwide species of cyanobacteria, is frequently related to toxic water blooms. These blooms have occurred in many countries, such as the Philippines [1], Australia [2], the United States of America [3], Canada [4] and Brazil [5], causing water contamination, risk of toxicity for hemodialysis and fishing prohibition. Moreover, numerous cases of animal poisoning due to cyanotoxins have been documented worldwide (livestock stupor in Australia [6], fish deaths in the U.K. [7] and cattle deaths in Switzerland [8]). Nevertheless, the most serious case of human poisoning attributed to cyanotoxins in drinking water, called “Caruaru syndrome”, occurred in Brazil, in 1996, when 76 patients died after hemodialysis treatment [9,10]. The deaths were attributed to the cyanotoxins microcystin and cylindrospermopsin.

*M. aeruginosa* is able to produce the hepatotoxin microcystins [11], which are members of a remarkable family of more than 90 cyclic peptides that inhibit hepatic serine/threonine protein phosphatases PP1 and PP2A of terrestrial mammals [12]. Furthermore, it is well-known that environmental factors – such as temperature, light intensity, nutrients, salinity, pH – and culture age can induce changes in toxicity and toxin concentration [13-15]. Despite the *M. aeruginosa* genome having already been completely sequenced, the cyanobacteria metabolic pathway of microcystin production has not been completely elucidated [16]. Otherwise, the microcystin biosynthetic gene cluster has already been characterized in *Microcystis*[17], *Planktothrix*[18], and *Anabaena*[19]. In this field, a proteomic approach could help to shed some light on toxin production. Nevertheless, only a few studies of cyanobacteria have been conducted using proteomic tools, being basically restricted to the *Synechocystis* species [20-24]. Furthermore, most of these studies focus on a specific compartment, such as the thylakoid membrane [23], outer membrane [21], plasma membrane [22], periplasm and cytoplasm [25,26]. Other cyanobacteria species, however, have also been proteomically considered, such as *Anabaena variabilis*[27], *Anabaena* sp. PCC 7120 [28,29], *Nostoc punctiforme*[30,31], Nevertheless these studies have been restricted to structural proteomic descriptions.

Otherwise, comparative proteomics have also been used to find differentially expressed proteins of cyanobacteria in response to environmental stresses like salinity [32,33], light [34,35], temperature [36], and pH [37]. Some studies have highlighted cyanobacterial changes becoming adapted to the symbiosis state [38,39]. However, the majority of studies have been done with *Synechocystis* sp. PCC 6803. Environmental conditions, like higher or lower salinity, acidity, variation of CO_2_ and Fe^2+^ concentration are directly related to cyanobacteria development in the natural environment. Therefore, it is important to perceive how cyanobacteria respond to these environmental changes to better understand the bloom formation processes.

Considering the enormous worldwide importance of *M. aeruginosa* in relation to freshwater quality, especially for public supply in reservoirs, it is interesting to note the low-quantity of research in proteomics [40,41] with comparative proteomics related to microcystin biosynthesis. To this end, this work aims to compare one naturally toxin-producing strain of *M. aeruginosa* with another naturally non-toxin producing strain using proteomic tools in order to better understand toxin production.

## Results and discussion

### Growth curves and microcystin detection

Aiming to compare two *M. aeruginosa* strains according their ability to produce cyanotoxins, both were growth in liquid medium according description previously described in literature. Although the differences are slight, there is correspondence between curves shown regardless of the wavelength used (Additional file 1: Figure S1). Microcystins are continuously produced during the cyanobacterial life cycle, but large quantities are commonly found at the end of log phase. For this reason, protein collection occurs at this growth point (Additional file 1: Figure S1, arrows) in which intracellular microcystin concentration is typically higher as previously observed for *M. aeruginosa* PCC 7820 [42].

**Figure 1:**
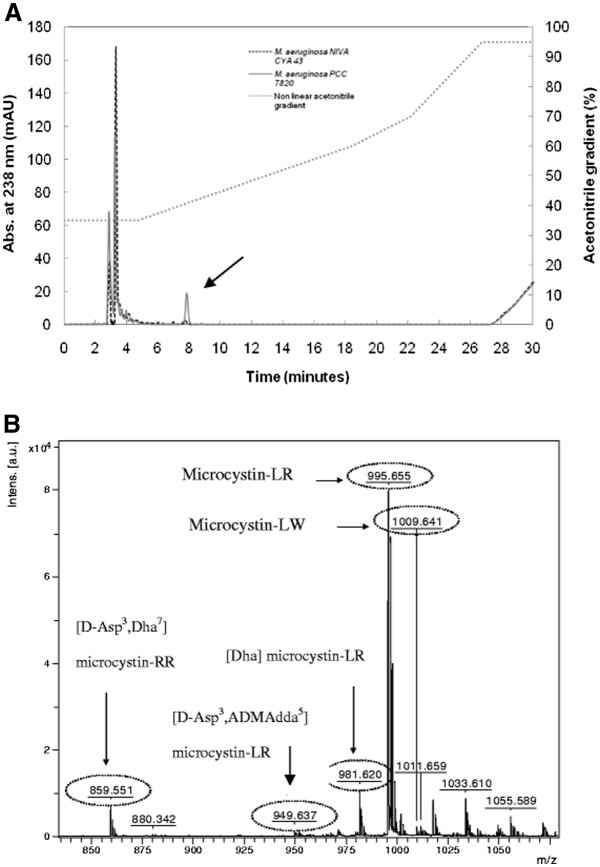
**(a) *M. aeruginosa *sample analysis in HPLC – reverse phase C18 column – and detection in 238 nm, following a non-linear gradient from 35% to 95% of acetonitrile/TFA 0.1%.** The fraction indicated by an arrow was evaluated by MALDI-TOF/TOF. (**b**) Mass spectra obtained by MALDI-TOF/TOF from *M. aeruginosa* PCC 7820 extract eluted fraction in HPLC and respective microcystin variant identifications.

In order to confirm if the *M. aeruginosa* strain PCC 7820 is really toxin producing and also if NIVA CYA 43 is really a non-toxin producing strain, the presence of microcystins in both cultures were analyzed in C18 column reversed-phase HPLC [Figure 1a]. The fraction indicated by the arrow was eluted only from the *M. aeruginosa* PCC 7820 extract after 7 min 48 s, with 40.7% acetonitrile. This fraction was concentrated, resuspended in a saturated solution of α-cyano-4-hydroxycinnamic acid matrix and analyzed by MALDI-TOF/TOF. The MS spectra showed that the fraction contained at least three different variants of microcystins [Figure 1b]. Microcystin-LR (*m/z* 995) was the variant with the most intensive ions. The other detected ions were identified as [Dha]microcystin-LR (*m/z* 981), [D-Asp^3^, Dha^7^microcystin-RR, microcystin-LW and [D-Asp^3^, ADMAdda^5^microcystin-LR, according to MS data comparison in the literature [43-45].

The observation of more than one microcystin variant was not surprising, because several cyanobacteria may be able to produce more than one microcystin variant [14]. Besides, in 50 to 75% of cyanobacterial blooms, toxicity is associated with the simultaneous production of several cyanotoxins [46].

### Proteome of *M. aeruginosa*

**Figure 2:**
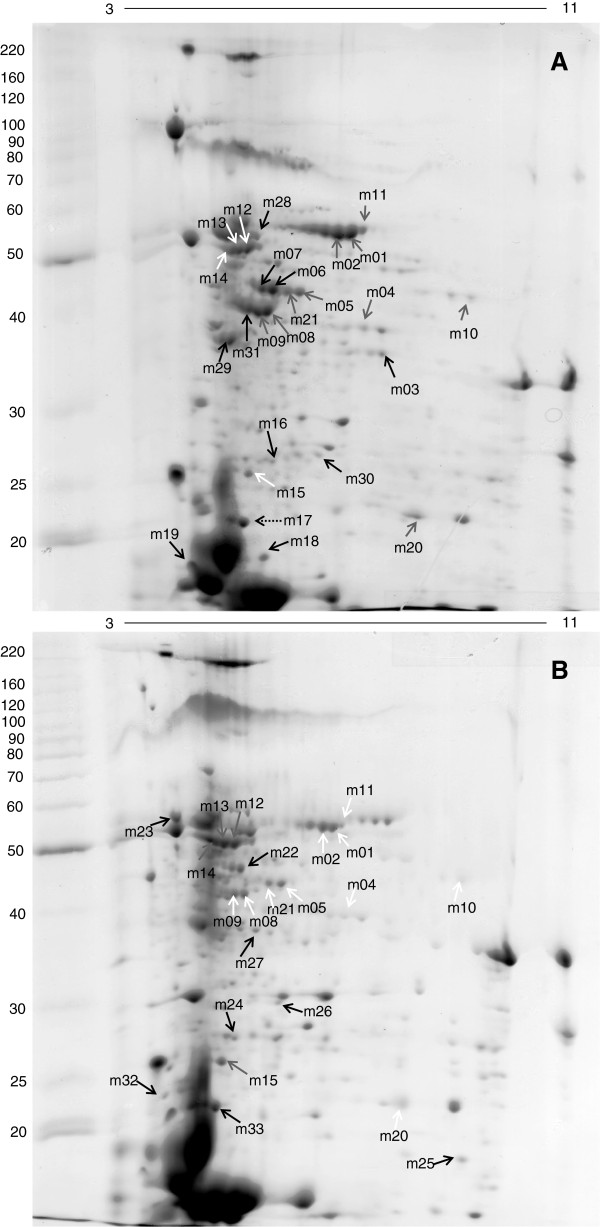
**(A) Proteomic map of *M. aeruginosa *PCC 7820. Gel 12.5%, stained with Coomassie, and pI 3–11.** (**B**) Proteomic map of *M. aeruginosa* NIVA CYA 43. Gel 12.5%, stained with Coomassie, and pI 3–11. Grey, white and black arrows indicate, respectively, up-regulated proteins; down-regulated proteins and proteins exclusive to that strain. Dotted arrow was the spot control.

SDS-PAGE (Additional file 2: Figure S2) was used to evaluate the protein extraction protocol quality, the reproducibility of the protein preparations, and to quantify the protein extracts for preparative 2-D gel analyses. For 2-DE, each strain was cultured in triplicate. From each biological replication, proteins were extracted from the whole-cell lysate. The *M. aeruginosa* PCC 7820 sample showed well-resolved protein bands with molecular masses between 10 and 116 kDa. Similar data was obtained from the *M. aeruginosa* NIVA CYA 43 sample (Additional file 2: Figure S2). In order to better investigate differential protein abundance visualized by SDS-PAGE, 2-DE analyses were performed. Protein extraction followed the same protocol used for SDS-PAGE, with the addition of a cleaning-up step, as described in the methodology. About 220 protein spots became visible and were clearly resolved in *M. aeruginosa* PCC 7820 2-DE [Figure 2a] and about 202 protein spots in *M. aeruginosa* NIVA CYA 43 2-DE [Figure 2b]. The majority of spots were observed between pH 4 and 7, as previously described for *Anabaena variabilis*[27], *Synechococcus* sp. PCC 7942 [47], and for *Synechocystis* sp. PCC 6803 [23]. This was the protein pattern expected for cyanobacteria samples, although most cyanobacterial proteomic studies have used strips with narrower pI ranges (4–7) and/or subproteomes from fractionation methods [20,22,23,35,37,39,40,48]. These studies have provided valuable information, but for *Microcystis,* we thought it more appropriate to expand the pI range and protein solubility (by adding Triton-X-100) to provide a more global profile, considering that this is the first report that compares one naturally toxin-producing strain with another naturally non-toxin-producing strain of *M. aeruginosa*.

### Identification and functional classification of the differentially expressed proteins

BioNumerics, software version 4.6, was used to analyze gel images in order to detect and select spots of interest. The five gel images obtained for each strain (biological replicates) were compared. At the end of the analysis, the software had provided volume values for each spot detected. With this information, linear regression analyses were done, and thus r^2^ was calculated. The linear regression presented r^2^ = 0.75 for *M. aeruginosa* PCC 7820 quintuplicates and r^2^ = 0.70 for *M. aeruginosa* NIVA CYA 43. Linear regression analyses of technical replicate gels commonly showed correlations higher than 0.80 [49,50]. It is important to highlight that in this study, we worked with biological replicates, and therefore the r^2^ value was slightly lower. Theoretical Mr and pI were obtained from MASCOT analysis and compared with the experimental values that were estimated directly from the gel images, using BioNumerics software. Moreover, toxin-producing cyanobacterial protein spots were considered differentially expressed when present/absent in all replicates: when at least two-fold changed (up or down) compared to control (non-toxin-producing) and also when the standard deviation bars did not intercross. There was a well-defined correlation between the theoretical and experimental Mr values as shown in Table 1.

**Table 1:** **Differentially expressed proteins identified from cellular extracts of axenic cultures of *M. aeruginosa *after tryptic digestion and MALDI-TOF/TOF analysis**

**Spot n.**	**Sequence**	**Accession no**	**Species**	**Protein ID**	**Sequence coverage (%)**	**Mowse score**	**Function**	**Theoretical pI/Mw (kDa)**	**Experimental pI/Mw (kDa)**
m01	KTFQGPPHGITVERD	166367530	*M. aeruginosa*	Ribulose bisophosphate carboxylase, major subunit	5.0	82	CO_2_ fixation (Calvin cycle) and photorespiration	6.8 / 53.20	6.4 / 53.86
	GIIIHIHR								
m02	KTFQGPPHGITVERD	166367530	*M. aeruginosa*	Ribulose bisophosphate carboxylase, major subunit	9.0	92	CO_2_ fixation (Calvin cycle) and photorespiration	6.8 / 53.20	6.2 / 53.84
	AVTMGFVDLMR								
	KTFEGPPHGIQAERD								
m03	KIAAEFPDNFRL	166363998	*M. aeruginosa*	Ferredoxin-NADP oxidoreductase	5.5	44	Oxidation and reduction (ficobilissome, thylakoid membrane)	6.1 / 44.81	6.9 / 35.61
	IAAEFPDNFR								
m04	KGILEYNDLPLVSSDYRG	166365244	*M. aeruginosa*	Glyceraldehyde-3-phosphate dehydrogenase	5.3	101	Glucose metabolic process (phosphorilation)	6.3 / 36.64	6.6 / 38.49
m05	RKPTGEILAISRI	166365988	*M. aeruginosa*	Fructose-1,6-bisphosphate aldolase class II (FBA)	49	74	Glycolysis	6.0 / 39.15	5.6 / 43.01
	YAGENFLRI								
	RKPTGEILAISR								
	KYAGENFLRH								
m06	KNIADHVAVEAMRE	166365743	*M. aeruginosa*	D-Frutose-1,6-bifosfatase classe II / sedoheptulose 1,7-bisfosfatase (FBPase classe 2/SBPase)	10.2	78	Carbohydrate biosynthesis (Calvin cycle)	4.7 / 37.66	5.1 / 42.99
	SIEELVVVVMDRPR								
	FFHGGAR								
m07	LQEMGITNPDR	166365743	*M. aeruginosa*	D-Frutose-1,6-bifosfatase classe II / sedoheptulose 1,7-bisfosfatase (FBPase classe 2/SBPase)	6.7	59	Carbohydrate biosynthesis (Calvin cycle)	4.37 / 37.12	4.95 / 43.12
	FVDTVHLFDQPK								
m08	RKPDFSAYIDPQRQ	159027237	*M. aeruginosa*	Phosphoribulokinase (PRK)	18.0	107	Carbohydrate metabolic process	5.45 / 38.04	5.00 / 40.88
	KVIVIEGLHPLYDERV								
	RKPDFSAYIDPQRQ								
	KVIVIEGLHPLYDERV								
m09	RKPDFSAYIDPQRQ	159027237	*M. aeruginosa*	Phosphoribulokinase (PRK)	27.3	112	Carbohydrate metabolic process	5.45 / 38.03	4.86 / 40.77
	KVIVIEGLHPLYDERV								
	RGHTYDDVMAAINSRK								
	RKPDFSAYIDPQRQ								
	KVIVIEGLHPLYDERV								
	VLSVLLGMTIGQIHR								
m10	TLPDLIHSAPR	196257787	*Cyanothece* sp.	FAD-dependent pyridine nucleotide-disulphide oxidoreductase	2.6	25	Oxidoreductase	6.41 / 46.87	8.41 / 42.98
m11	KTFQGPPHGITVERD	166367530	*M. aeruginosa*	ribulose bisophosphate carboxylase, major subunit	10.6	91	CO_2_ fixation (Calvin cycle) and photorespiration	6.76 / 53.20	6.57 / 54.67
	RGITMGFVDLMRE								
	KNHGIHFRV								
	TFQGPPHGITVER								
m12	RDNDVTIDDLFYRA	166365540	*M. aeruginosa*	Porin type major outer membrane protein	15.7	106	Transport (integral membrane protein)	4.14 / 60.14	4.78 / 50.13
	KFADINFVYVRS								
	RLQAGNFNNTFNQSGPTRT								
	KFADINFVYVRS								
	LQAGNFNNTFNQSGPTR								
	QSQTPAHQSRANQR								
m13	RDNDVTIDDLFYRA	166365540	*M. aeruginosa*	Porin type major outer membrane protein	10.4	96	Transport (integral membrane protein)	4.14 / 60.14	4.64 / 50.1
	LQAGNFNNTFNQSGPTR								
	RDVSPTAWAYEALRS								
	KFADINFVYVRS								
m14	LQAGNFNNTFNQSGPTR	166365540	*M. aeruginosa*	Porin type major outer membrane protein	8.6	84	Transport (integral membrane protein)	4.14 / 60.14	4.48 / 50.43
	KFADINFVYVRS								
	RLQAGNFNNTFNQSGPTRT								
m15	RGWINDINETQNTTVNYPIIADGDRK	166366392	*M. aeruginosa*	Peroxiredoxin	19.4	180	Cell redox homeostasis	4.17/16.55	4.68 / 25.39
	KVIALSVDSAESHRG								
m16	QYFGETDETVNLR	166363870	*M. aeruginosa*	Triosephosphate isomerase	12.2	62	Triosephosphate isomerase activity (glyconeogenesis and glycolysis)	4.14 / 25.21	4.85 / 27.01
	RQYFGETDETVNLRV								
m17	RPDYISDFLTK	166368140	*M. aeruginosa*	Superoxide dismutase (SOD)	9.0	40	Catalyzes of superoxide to peroxide and molecular oxygen	5.83 / 21.72	4.6 / 21.50
	YLDYQNR								
m18	IVQASTGLEVLSDSILVQKLR	220908731	*Cyanothece* sp. PCC 7425	ABC transport related	5.8	78	Stress response	6.07 / 64.52	4.99 / 18.61
	FLIATPLLLALMR								
m19	RLITYGVVAGDVTPIEEIGLVGVRE	166363768	*M. aeruginosa*	Allophycocyanin alpha subunit	25.5	104	Photosynthesis (phycobilisome)	4.21 / 17.19	3.79 / 18.09
	YLSPGELDR								
	DMDYYLR								
m20	RVPAEIVFDQGLGDLFVCRV	166364254	*M. aeruginosa*	Carbonic anhydrase, beta-type (CA)	8.3	130	Carbon utilization	8.45 / 26.10	7.50 / 21.93
m21	KYAGENFLRH	166365988	*M. aeruginosa*	Fructose-1,6-bisphosphate aldolase class II (FBA)	10.0	59	Glycolysis	6.00 / 39.16	5.39 / 43.1
	RKPTGEILAISRI								
	RKPTGEILAISRI								
m22	RSIEELVVVVMDRPRH	166365743	*M. aeruginosa*	D-fructose 1,6- bisphosphatase class 2/sedoheptulose 1,7- bisphosphatase (FBPase classe 2/SBPase)	14.2	67	Carbohydrate biosynthesis (Calvin cycle)	4.37 / 37.68	4.95 / 45.86
	FFHGGAR								
	FVDTVHLFDQPK								
	RFVDTVHLFDQPKY								
m23	DVSPTAWAYEALR	166365540	*M. aeruginosa*	Porin type major outer membrane protein	4.6	45	Transport (integral membrane protein)	4.14 / 60.14	3.94 / 57.46
	DVERALTAPHLTR								
m24	RQYFGETDETVNLRV	166363870	*M. aeruginosa*	Triosephosphate isomerase	6.5	62	Triosephosphate isomerase activity (gluconeogenesis and glycolysis)	4.14 / 25.21	4.85 / 27.01
	RQYFGETDETVNLRV								
m25	RTFTEVAPQPAPEPSVSPIRG	166367494	*M. aeruginosa*	Hypothetical protein MAE_47530	30.8	69	Non function related	5.32 / 13.01	8.39 / 17.08
	RGEPQLNPGDYVLGRV								
m26	RAPYDESEVVYHLDLYENKG	159029497	*M. aeruginosa*	Phycobilisome rod linker polypeptide	2.1	119	Photosynthesis (phycobilisome)	9.24/ 32.21	5.51 / 29.88
	FQQCLVQTR								
	RETPVMSQAEIHSR								
m27	LVSLGLLK	159028959	*M. aeruginosa*	Cobalt ABC transporter, inner membrane subunit CbiQ	3.1	72	Cobalt trasnport and biosynthetic process of cobalamin	10.22/28.53	5.18 / 37.08
m28	KKILLLLVLIVAVLNFGKT	15642898	*T. maritima*	Zinc ABC transporter, periplasmic zinc-binding protein	7.1	61	Metal transport	6.25 / 30.12	4.85 / 52.85
m29	KPDFSAYIDPQR	166366586	*M. aeruginosa*	Phosphoribulokinase (PRK)	3.6	42	Kinase and transferase	5.12 / 37.85	4.42 / 36.73
							ATP binding		
m30	QAGSIVCISSISGDR	166367746	*M. aeruginosa*	PHA-specific acetoacetyl-CoA reductase	6.3	49	Oxidoreductase	5.66 / 25.18	5.89 / 27.19

After the BioNumerics software analyses, 30 proteins of interest were chosen for identification based on differential expression. These proteins were identified by MASCOT and corresponded to 21 different proteins classes [Table 1]. Almost all identified proteins showed sequence correspondence to *M. aeruginosa*. The only 3 proteins which did not correspond to *M. aeruginosa* were m10, m18, and m28; all of which showed similarities to other cyanobacteria. One spot (m17), identified as superoxide dismutase, was used as a control to increase MS data reliability. Protein identifications showed that 61% of differential proteins are related to energy metabolism, 18% corresponded to membrane proteins, 6% are carboxysome and periplasmic proteins, 12% are cytoplasmic proteins and 3% are hypothetical proteins. To avoid double counting, rubisco was considered only in energetic metabolism proteins, although this enzyme is located inside carboxysome. Four of these 21 proteins were found in more than one spot on the gel. Among these are the enzymes Rubisco (spots m01, m02, m11), FBA class II (spots m05, m21), PRK (spots m08, m09, m29), and porins (spots m12, m13, m14, m23) [Table 1].

### Energy metabolism proteins

**Figure 3:**
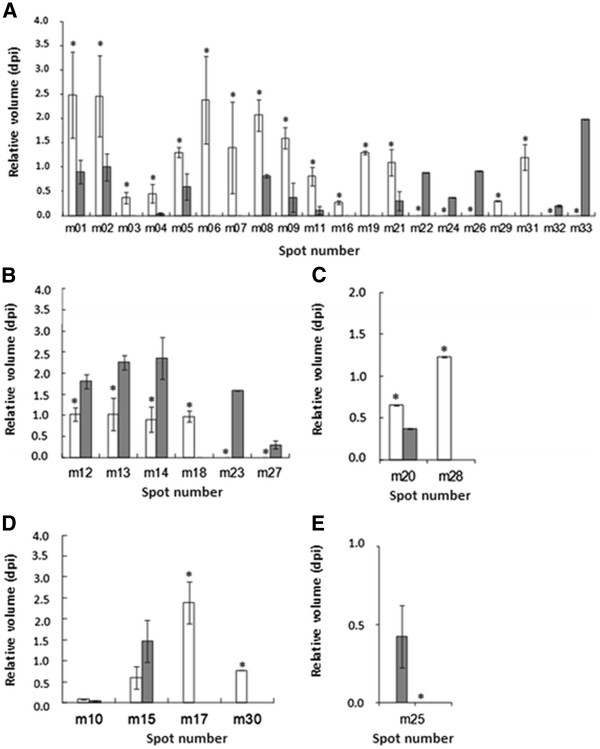
**Differentially expressed proteins obtained from 2D gels of *M. aeruginosa *PCC 7820 (white) and NIVA CYA 43 (grey) strains related to (**a**) energy metabolism; (**b**) membrane proteins (external and internal); (**c**) periplasmic proteins; (**d**) cytoplasmic proteins and (**e**) hypothetical proteins.** Vertical bars correspond to standard deviation. The asterisks indicate protein expression that differed significantly (p < 0.05) between the toxin producing (PCC 7820) and non-toxin producing (NIVA CYA 43) strains by the Student’s t-test.

It is interesting to observe that most of the differentially expressed proteins identified are related to energetic metabolism, and these proteins were up-regulated in the toxin-producing strain. It is important to declare here that we did not estimate the amount of new protein synthesis, but the protein abundance. This increased protein amount can result from higher synthesis (up-regulation) and/or stabilization of the identified protein. Ribulose bisphosphate carboxylase (rubisco), glyceraldehyde-3-phosphate dehydrogenase (GAPDH), triosephosphate isomerase, D-fructose 1,6-biphosphatase class II/sedoheptulose 1,7-biphosphatase (FBPase class II/SBPase) - fructose-1,6-biphosphate aldolase (FBA), phosphoribulokinase (PRK), ferredoxin-NDP^+^ oxidoreductase (FNR) were all up-regulated in the toxin-producing strain in comparison with the non-toxin-producing strain [Figure 3a]. Rubisco, GAPDH, PRK and FNR were also observed in thylakoid membrane of *Synechocystis* sp. PCC 6803 the first time that thylakoid membranes from this cyanobacteria were used in proteomic studies [23]. Rubisco protein appears as isoform in 3 different spots with pI variation in the toxin-producing strain. FBA class II protein also appears in 3 different spots, with pI variation in the toxin-producing strain. However, this protein appears in only two different spots in the non-toxin-producing strain. The same result was observed for the PRK protein, which appears in 3 different spots with pI variation. Nevertheless, the toxin-producing strain presented the same protein at an extra spot, with a molecular mass below that of the other two.

The proteins identified here are all involved in the Calvin cycle, glycolysis and respiration: processes responsible for providing energy to the cell. In the toxin-producing strain, rubisco (m01, m02 e m11), FBPase class II/SBPase (m06, m07 e m22), FBA (m05 e m21) and PRK (m08, m09 e m29) presented relative intensities of expression more than twice those seen in the non-toxin-producing strain. GAPDH (m04) was ten times higher in the toxin-producing strain. FNR was identified only in the toxin-producing strain. Therefore, it is suggested that overexpression of proteins involved in energy production by toxin-producing PCC 7820 probably means a compulsory requirement to produce microcystin, and this production may be synonymous of higher energy consumption. This has already been noted in studies with *Streptococcus suis*[51], *Candida glabrata*[52] and *Yersinia pestis*[53], which showed that expression modifications in metabolic proteins suggest higher virulence. The proteome showed an increase in expression in metabolism-related proteins, like fructose bisphosphate aldolase, glycerol-3-phosphate dehydrogenase and triosephosphate isomerase. According to these authors, an increase in glycolytic metabolism suggests higher virulence. Besides, changes in central carbon metabolism have been associated with virulence attributes and the inactivation of virulence regulators in *C. albicans*[54]. Proteomic results with the bacteria *Yersinia pestis* virulence indicate that expression changes in metabolic proteins may have a more significant role in virulence than was previously appreciated [53]. In the present study, it also seems that proteins involved in the Calvin cycle, respiration, and glycolysis play important roles related to microcystin production, since the Calvin cycle is the pathway most activated in the toxin-producing *M. aeruginosa* PCC 7820.

### Carboxysome and periplasmic proteins

Carbonic anhydrase (CA) and a protein with high sequence similarity to zinc ABC transporter of *Thermotoga maritima* MSB8 were identified and both were up-regulated by the toxin-producing strain [Figure 3c and Table 1. The up-regulation of CA may occurs at the same time as the expression of zinc ABC transporter at the toxin-producing strain, since zinc is a metal required for the active site of CA, where this metal has a central role in carbon fixation for photosynthesis [55].

These results are in accordance with observations about energy metabolism proteins. CA is an enzyme that participates in the CO_2_ concentrate mechanism (CCM). In aquatic environments with pH values above 7, inorganic carbon is available preferably in the form of carbonic acid (HCO_3_^-^) [56]. Therefore, aquatic photosynthetic microorganisms developed CCM to generate an intracellular pool of inorganic carbon.

Data reported here shows that CA was expressed by the toxin-producing PCC 7820 [Figure 3c], 1.8 times stronger than the non-toxin-producing strain. Probably, the toxin-producing strain has an improvement of carbon fixation. This improvement could be directly related to microcystin production, since the use of carbons and energy was necessary in this process. Moreover, this result could also be related to an increase of metabolic flux through the Calvin cycle, mentioned above. In both cases, this reflects a more efficient carbon fixation that leads to PHA accumulation. With a higher quantity of carbon, it may be possible to maintain an efficient microcystin production.

### Cytoplasmic proteins

The identified protein in spot m10 presented a high similarity with an enzyme from the flavoprotein family: a FAD-dependent pyridine nucleotide-disulphide oxidoreductase, present in *Cyanothece* sp. This enzyme acts in cellular redox homeostasis processes and oxidation and reduction, where its molecular function is to carry out the activities of oxidoreductase and the electron carrier. Co-factors iron (Fe) and sulfur (S) are necessary for its action [57]. Flavoproteins seem to play an important role in bloom situations since they are frequently found in cyanobacteria collected from dense blooms. In environments with dense blooms, Fe and copper deficiency often occurs [57]. Flavoproteins have the capacity to substitute ferredoxins in some cyanobacteria when they lack Fe, because these two protein classes are similar in relation to charge set and redox properties [58]. This protein was expressed by both strains at similar statistical levels [Figure 3d] suggesting that both cyanobacteria may naturally have this advantage. Therefore, their growth may be favored in these environments, but toxin production may not necessarily be favored.

Peroxiredoxin (m15), observed only in the *M. aeruginosa* PCC 7820 gel, presents physiological significance as anti-oxidative stress in cyanobacteria [59]. This enzyme catalyzes the reduction of various hydroperoxides [60] and seems not to be related to microcystin production. Otherwise, the tryptic fragments of protein spot m17 were identified as superoxide dismutase, an enzyme that protects the cell against damage caused by superoxide radicals [61].

The PHA-specific enzyme acetoacetyl-CoA reductase (m30) was exclusive to the toxin-producing strain. In *Synechocystis* sp. PCC 6803, this protein has the function of PHA (polyhydroxyalkanoate) accumulation in the cell and, together with ketothiolase-β PHA specific enzyme, forms the first complete PHA biosynthesis route known in cyanobacteria [62]. PHAs are biodegradable polyesters, carbon and energy storage compounds, which are deposited as insoluble inclusions at cytoplasm [62]. The fact that this protein appeared only in the toxin-producingstrain indicates that to maintain the state of microcystin producer, *M. aeruginosa* PCC 7820 must have access to energy reserves. It may be considered that, since the toxin-producing strain has higher expression levels of proteins related to energy metabolism in relation to the non-toxin-producing strain, there is a need to produce proteins required for intracellular accumulation of energy produced. The expression of this enzyme only by the toxin-producing stain suggests that producing microcystin means that more energy is spent due to the carbon and energy accumulation need seen in toxin-producing *M. aeruginosa* PCC 7820.

### Membrane proteins

In this work, 4 spots were identified as external membrane porins. The porin protein appears in 4 different spots on the non-toxin-producing strain gel, with 3 isoforms and another spot with pI and MW different from the other three. Surprisingly, spots m12, m13 and m14 were about twice as highly expressed in the non-toxin-producing in relation to the toxin-producing strain and spot m23 was exclusive to the non-toxin-producing strain [Figure 3b]. This observation represents an inversion of what has been seen for the other protein classes, and no toxin relation has been drawn until now. However, higher expression of porin in the non-toxin-producing strain seems to be in accordance with higher expression of peroxiredoxin. In relation to phycobilisome proteins, we identified allophycocyanin alpha subunit (m19) only in the toxin-producing strain, phycobilisome rod linker polypeptide (m26) and phycocyanin alpha phycocyanobilin lyase related protein (m32), of which the last three only appear in the non-toxin-producing strain.

Spot m18 appeared only in the toxin-producing strain [Figure 3b] and presented high sequence similarity with an ABC transporter of *Cyanothece* sp. PCC 7425. ABC transporters are a family of membrane proteins that carry out the transport of molecules rather than ions, and are involved in several physiological processes [63].

Otherwise, the ABC transporter was considered as a stress-related protein [36,64]. In *Spirulina platensis*, this protein was detected in all subcellular fractions up-regulated when this cyanobacterium was submitted to high-temperature [64] and to low-temperature stresses [36] and its transcriptional expression pattern was well correlated with the protein expression pattern.

Then, Amnuaykanjanasin and Daub [65] recently observed that the fungal *Cercospora nicotianae* disrupted at the ABC transporter gene (*atr*1) shows a dramatic reduction in the production of the toxin cercosporin. These data [65] indicate that qualitative and quantitative differential expression of proteins in the mycelium provides evidence to partly explain the mechanism of pathogen virulence differentiation. Similarly, our results indicate that the toxin-producing *M. aeruginosa* PCC 7820 presents higher synthesis of proteins involved in metabolism in relation to the non-toxin-producing *M. aeruginosa* NIVA CYA 43.

## Conclusions

**Figure 4:**
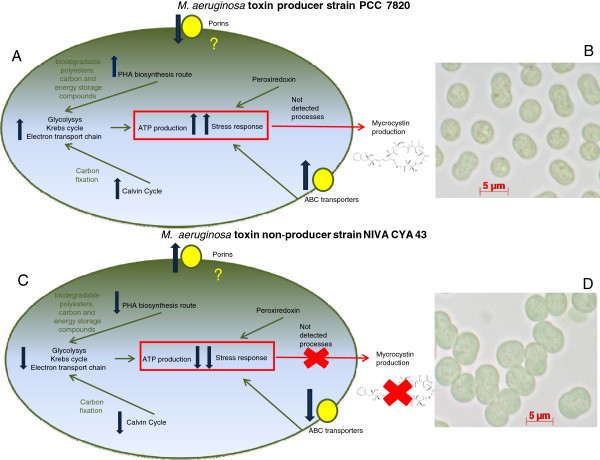
**Schematic representation of cellular differences observed in this report at proteomical level, between *M. aeruginosa *PCC 7820 (**A**) and NIVA CYA 43 strains (**C**).** Light microscopy images of *M. aeruginosa* PCC 7820 (**B**) and NIVA CYA 43 strains (**D**) were also provided. Blue arrows represent up- or down regulation processes observed. Yellow forms indicated the membrane proteins identified in this report.

It have been observed that microcystin production provides a photosynthetic and ecological advantage – adjusting rubisco synthesis [66], participation in processes associated with light reactions [67] and predation inhibition [68,69]. Putative functions of microcystin should result in a competitive advantage for those strains that are able to produce it. Therefore, microcystin non-toxin-producing strains need to develop compensating mechanisms. According Meiβner et al. [70], non-toxic strains have genes coding for proteins involved in toxin production (peptide synthetases). Nevertheless, those strains seem to produce fewer gene copies in relation to toxics trains, mostly differing by genes content. Data reported here show that toxic strains are capable of producing proteins involved in energy metabolism at a higher quantity than nontoxic strains. However, in competition experiments, it was shown that nontoxic strains are better competitors for light than toxic strains [71]. These authors also suggest that non-toxic strains may invest their resources in other cellular functions since they are unable to utilize energy to produce microcystin. Our results point in same direction. Nontoxic strains probably have compensating mechanisms, using energy not spent in microcystin production in multiple cellular functions, like higher porin production and also in the improvement of cell size. Data reported here suggest a dualistic response: either microcystin stimulates the production of metabolism-related proteins or else metabolism proteins are expressed in higher quantities that enable the cell’s microcystin synthesis. In summary, energetic metabolism proteins (Calvin cycle, glycolysis and respiration) were observed, being expressed in larger amounts by the toxin-producing strain (*M. aeruginosa* PCC 7820). Corroborating these data, the expressions of other proteins that depend on energy production, which includes proteins related to carbon and nitrogen accumulations forming a kind of strategic cellular reservoir for eventual nutrient starvation, were also obtained. In an overview, the energy improvement observed in toxin production may provide a continuity of microcystin production under different environmental conditions. Additionally, the same proteins involved in stress response also seem to be linked to microcystin production, which included the ABC transporters and as well as the peroxiredoxin forms. This information leads to suggest the idea that an improvement of carbon and nitrogen fixation is necessary in the first moment. This modification may lead, in the next moment, to a higher ATP synthesis [Figure 4. In parallel, the necessity of a stress response signal was also observed, correlated with the enhanced expression of cytoplasmic and membrane proteins. These processes together seem to orchestrate part of the microcystin synthesis process. Moreover, it is very interesting to notice that the *Microcystis*-cell machinery may work in a complex and harmonious manner, having the modulation of several proteins involved in microcystin production. Despite findings in energy metabolism and stress response provided here, it is also important to be clear that other processes could also be involved in cyanotoxin production that were not detected here by proteomic techniques. Another important issue is that we do not know at the moment if the non-toxin producer has the microcystin synthetase (mcy) genes that encode specific peptide synthetases, which have been shown to be involved in the construction of the toxic peptides. Nevertheless, studies performed by Cristiansen et al. [72] showed that gene cluster deletion is a rare event in nontoxic cyanobacteria. These modifications associated with the evolutionary diversification of the strain lineages lead to a combination of cyanobacteria that either lost or still contain the mcy-gene cluster in the same environment. Overall, proteomics technology may be considered a powerful tool used for uncovering the nature of microcystin production and its regulation in function of environmental changes. This understanding may significantly contribute to the development of compounds which may be employed directly in the environment, aiming to block microcystin production by a toxin-pathway-specific means, thus preventing its release into water.

## Methods

### Organisms and culture conditions

*M. aeruginosa* PCC 7820 and *M. aeruginosa* NIVA CYA 43 were acquired from CSIRO (Commonwealth Scientific and Industrial Research Organization), Australia, and grown in a modified MLA medium (MgSO_4_.7H_2_O 200 mM, NaNO_3_ 1 M, K_2_HPO_4_ 40 mM, H_3_BO_3_ 40 mM, NaHCO_3_ 200 mM, CaCl_2_.2H_2_O 200 mM, vitamins and micronutrients, pH = 7.5) in 1000 mL Erlenmeyer flasks (800 mL liquid). The cultures were maintained at 22°C under a 12:12 h light/dark cycle and illuminated with 6 μmol of photons.m^-2^.s^-1^ of fluorescent white light. Cells were harvested from the growth medium at late exponential phase (OD_680nm_ = 0.7 – for *M. aeruginosa* PCC 7820, and OD_680nm_ = 0.8 – for *M. aeruginosa* NIVA CYA 43). The growth profile of *M. aeruginosa* was conducted in order to determine the end of the exponential phase. The growth curve was determined in 25 days, and the OD evaluation was conducted simultaneously every three days during this period. All measurements were done in triplicate at three different ODs: 680 nm, 730 nm and 750 nm for each sample, considering wave length absorbed by cyanobacterial pigments, such as chlorophyll and bilins. These three different ODs were used to verify the correspondence between growth curves obtained by these different wave lengths and if both strains grew equally well. For cell extraction, OD_680nm_ was used to track cell growth. Although there were slight differences, correspondence between curves was shown regardless of the wavelength used (Additional file 1: Figure S1). For protein extraction, cultures of *M. aeruginosa* PCC 7820 and NIVA CYA 43 were centrifuged at OD_680nm_ = 0.7 and OD_680nm_ = 0.8, respectively, corresponding to the end of log phase.

### Microcystin detection

During log phase, 1 mL aliquots of each strain were collected for evaluation. These aliquots were ground under liquid nitrogen (five times) followed by pelleting of insoluble debris by centrifugation at 12,100 g for 5 min. Protein concentration was determined by Qubit™ Fluorometer (Invitrogen, Oregon, USA). Each aliquot was separately analyzed on a reversed-phase C-18 HPLC (GE Healthcare) column (Vydac 218 PT 5 μm pore), at flow of 1 mL.min^-1^. This was run for 35 min and followed by a non-linear acetonitrile gradient (35% to 95%), using TFA 0.1% as ion pairing, according to Harada et al. [73] with modifications. The absorbance was monitored at 238 nm. The collected fractions were dried in speed vacuum (Labconco). Fraction analyses were conducted in a MALDI-TOF/TOF Ultraflex III (Bruker Daltonics). The chromatography fractions were resuspended in 20 μL TFA 0.1% and mixed in a 1:3 v/v proportion with a saturated solution of α-cyano-4-hydroxycinnamic acid matrix. From this mixture, 1 μL was manually spotted onto a MALDI target plate. The spectra were acquired in positive reflector mode. Ions with enough intensity were selected for cleavage by MS/MS. Microcystin fragmentation standard was generated by LIFT mode with external calibration using Peptide Calibration Standard II (Bruker).

### Protein extraction and two-dimensional gel electrophoresis

Cells were harvested by centrifugation (3,000 g for 30 min at 4°C) and mixed in buffer extraction (5 mM Tris–HCl containing 5 mM CaCl_2_.2H_2_O, 0.01% triton-X-100 and 1 mM proteinase inhibitor cocktail (GE Healthcare)), followed by grinding under liquid nitrogen (five times) and pelleting of the insoluble debris by centrifugation at 3,000 g for 30 min at 4°C. The protein concentration was determined using Qubit^TM^ Fluorometer (Invitrogen, Oregon, USA) according to the manufacturer’s instructions. 2-DE was performed with 13 cm gels with a 3–11 pH range loaded with 600 μg of protein. Prior to electrophoresis, protein samples (biological triplicate for each strain) were TCA precipitated Pellets were washed three times with 100% cold acetone and cleaned with 2-DE cleaning-up kit (GE Healthcare), as indicated by the manufacturer. Precipitate was solubilized in 250 μL of an electrofocusing solution containing 8 M urea, 2 M thiourea, 2% (v/v) CHAPS, 1% (v/v) IPG buffer 3–10, 65 mM DTT and traces of bromophenol blue. Strips were rehydrated in this solution overnight at 20°C. The isoelectric focusing of the proteins was performed on an IPGphor (GE Healthcare), and the running conditions were (i) 500 V for 1 h; (ii) 1000 V for 2 h; (iii) 3500 V for 5h30m and (iv) 3500 V for 10 h until a total of 40,000 Vh. The gel strips were then incubated in equilibrated buffer (Tris–HCl 1.5 M, 6 M urea, 30% (v/v) glycerol, 2% SDS, 1% DTT, 1% iodoacetamide and traces of bromophenol blue) for 30 min before being pointed at the top of a 12.5% polyacrylamide gel and sealed with 0.5% agarose. Electrophoresis was carried out at 15°C and 67 mA/gel for 8 h. Proteins were detected by Coomassie Blue G-250, scanned using an image scanner (HP scanner Scanjet 8290) and evaluated with the BioNumerics software 4.6 version (Applied Maths NV, Belgium) as described below.

### Image analysis

A total of five gel replicates for each strain (*M. aeruginosa* PCC 7820 and *M. aeruginosa* NIVA CYA 43) were selected and submitted to in silico analysis by BioNumerics 5.1 software (Applied Maths). 16-bit TIFF gel image replicates (gray scale and 600 dpi) were aligned and screened by the software in order to detect the protein spots and determinate their concentration, molecular weight (Mw) and isoelectric point (pI). Afterward, gel images of all strains were intercrossed and screened for spot differences and similarities. Statistic correlation analysis (R^2^) was applied to evaluate differential gel data. Correlations cut offs were applied and gels with R^2^ lower than 0.7 were discarded. Mean value of spot volume (dpi) from spot densities was used for further comparison of protein expression, taking into account spots with relative volume equal to or bigger than 0.1 dpi. Moreover, the toxin-producing cyanobacterial protein spots were considered differentially expressed when present/absent in all replicates; when at least two-fold changed (up or down) compared to control (non-toxin-producing strain); when the standard deviation bars did not intercross. Finally, all spots that achieved the minimum requires were submitted to Student’s *t*-test and differences with *p* < 0.05 were considered significant.

### In-gel digestion and MALDI-TOF analysis

In-gel digestion, including reduction with DTT and alkylation with iodoacetamide, was performed according to Schevchenko et al. [74], with minor modifications, using 600 ng of trypsin gold-mass spectrometry grade (Promega) per spot and digested for 24 h at 37°C. The samples were air dried, resuspended in 10 μL of milli-Q water and analyzed in a MALDI-TOF/TOF Ultraflex III (Bruker Daltonics). One μL of each digest was mixed with 3 μL of a saturated solution of α-cyano-4-hydroxycinnamic acid matrix and was manually spotted onto a MALDI target plate. The spectra were acquired in positive reflector mode with 200 random shoots. Ions with enough intensity were selected for cleavage by MS/MS. Fragmentation standard was generated by LIFT mode with external calibration, performed using Peptide Calibration Standard II (Bruker) with accuracy of 0.3 Da.

### Database search

Proteins were identified by using the MASCOT search engine (Matrix Science, London, UK) using the NCBInr database. The search parameters included carbamidomethylation of cysteines, oxidation of methionines, one miscleavage by trypsin and 80 ppm mass accuracy. The identification of the proteins was repeated at least once using spots from different gels. Identifications with probability based on Mowse Scores ≥ 50 were considered significant (P < 0.05), and identifications with Mowse Score < 50 were then disregarded.

## Abbreviations

ABC: ATP-binding cassette; Adda: (2S,3S,8S,9S)-3-amino-9-methoxy-2,6,8-trimethyl-10-phenyldeca-4,6-dienoic acid; CA: Carbonic anhydrase; CHAPS: [(3-Cholamidopropyl)dimethylammonio]-1-Propanesulfonic Acid; OD: Optic density; DTT: Dithiotreitol; FBA: Fructose-1,6-biphosphate aldolase; FBPase class II/SBPase: D-fructose-1,6-biphosphate classII/sedoheptulose 1,7-biphosphate; FNR: Feredoxin-NADP^+^ oxidoreductase; GAPDH: Glyceraldehyde-3-phosphate dehydrogenase; HCO_3_^-^: Carbonic acid; HPLC: High performance liquid chromatography; MALDI-TOF: Matrix assisted laser desorption ionization time of flight; NRPS: Non ribosomal peptide synthesase; PHA: Polyhydroxyalcanoate; pI: Isoelectric point; PKS: Polyketide synthase; PRK: Phosphoribulokinase; 2-DE: Bidimensional electrophoresis; DSD-PAGE: Sodium dodecil sulphate-poliacrylamide gel electrophoresis; SOD: Superoxide dismutase; Rubisco: Ribulose bisophosphate carboxylase; TCA: Trichloroacetic acid; Vh: Volt hours.

## Competing interests

The authors declare that they have no competing interests and declare no conflict of interest.

## Authors’ contributions

AT: participated on study design and all experimental procedures as well as the manuscript draft. BPA: participated on experimental design, execution and manuscript draft. WCA: participated on experiment execution. AM: participated on protein identification. BSM: participated on protein identification. OLF: performed the study design and coordinated the manuscript draft. All authors read and approved the final manuscript.

## Supplementary Material

Additional file 1 **Figure S1.** Growth curves of *M. aeruginosa* PCC 7820 (a) and *M. aeruginosa* NIVA CYA 43 (b). Cyanobacteria development was evaluated at wavelength of 680 nm, 730 nm and 750 nm. Black arrows correspond to the end of log phase in which protein extraction occurs. Click here for file

Additional file 2 **Figure S2.** SDS-PAGE 15% by using crude protein cytosolic extract (50 μg.ml^-1^) from *M. aeruginosa* samples. (a) corresponds to *M. aeruginosa* PCC 7820 and (b) corresponds to *M. aeruginosa* NIVA CYA 43. Gel was Coomassie stained. M corresponds to molecular mass. Click here for file
