# Supplementary figures and images for: Comparative proteomics between natural Microcystis isolates with a focus on microcystin synthesis
Source: Proteome Sci. 2012 Jun 7;10:38. doi: 10.1186/1477-5956-10-38 (PMC3522533; doi:10.1186/1477-5956-10-38)

**Supplementary Figure 1**


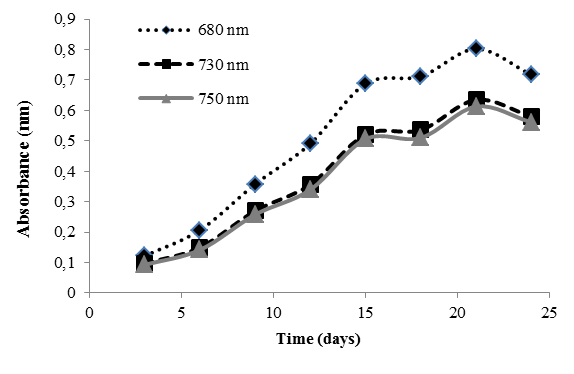


(a)


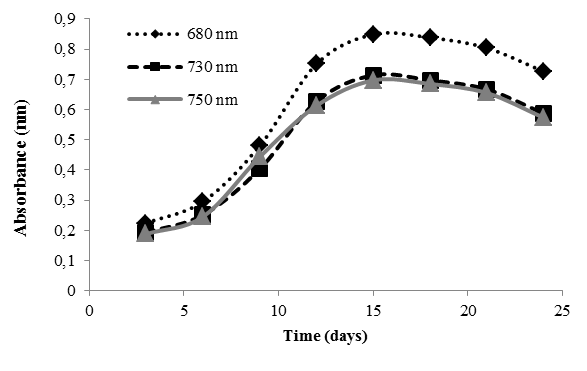


(b)

Supplement: Additional file 1 — Figure S1. Growth curves of M. aeruginosa PCC 7820 (a) and M. aeruginosa NIVA CYA 43 (b). Cyanobacteria development was evaluated at wavelength of 680 nm, 730 nm and 750 nm. Black arrows correspond to the end of log phase in which protein extraction occurs. [file 1477-5956-10-38-S1.doc]

**Supplementary Figure 2**

M

(a)

(b)B


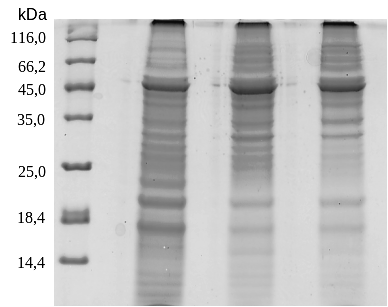

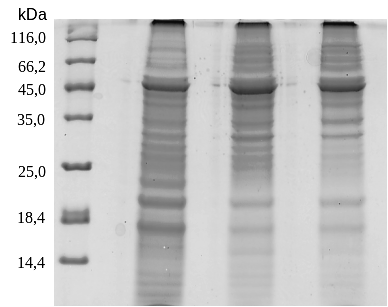

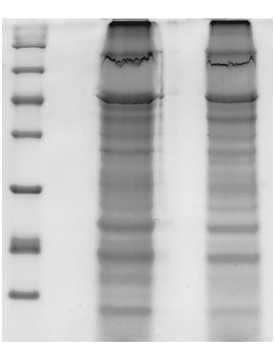

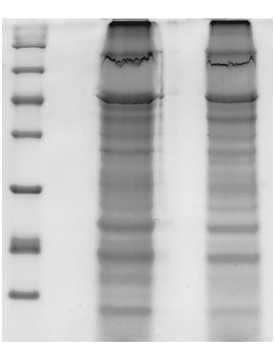

Supplement: Additional file 2 — Figure S2. SDS-PAGE 15% by using crude protein cytosolic extract (50 μg.ml-1) from M. aeruginosa samples. (a) corresponds to M. aeruginosa PCC 7820 and (b) corresponds to M. aeruginosa NIVA CYA 43. Gel was Coomassie stained. M corresponds to molecular mass. [file 1477-5956-10-38-S2.doc]
